# Observations on the Diagnosis between Central Stricture of the Stomach, and Some of the Abdominal Lesions with Which It May Be Confounded
*The earlier appearance of this paper has been prevented by the pressure of much more important articles for our Original Department.


**Published:** 1816-04

**Authors:** Shirley Palmer


### Medico-Chirurgical Journal and Review.

VOL. I.] APRIL, 1816. [NO. 4.

### PART I.

### ORIGINAL COMMUNICATIONS.

*Observations on the Diagnosis between central Stricture of the Stomach, and some of the abdominal Lesions with which it may be confounded.** By SHIRLEY PALMER, M. D.

IN resuming this subject, I do not profess to establish incontrovertibly the diagnosis of central gastric stricture, or contrast it with *all* the abdominal maladies for which, from the general and often close resemblance of its external phænomena, it may be mistaken. My sole object is to present a rude outline, which more accurate and extended observation may correct and fill up. Indeed, on re-examining the stock of materials, collected and partly arranged, three months since, for the composition of this paper, I find those belonging to myself so few and unimportant, that, had not a pledge for its appearance been then given,† a production, thus crude and imperfect, would certainly have never been obtruded on the public eye.

The abdominal lesions, which in their external charac-

* The earlier appearance of this paper has been prevented by the pressure of much more important articles for our Original Department.

† See page 17 of this volume.

ters present the closest resemblance to central stricture of the stomach, may be conveniently arranged under two classes. Those which implicate the structure of the stomach itself will form one class; and those which have their seat in the contiguous organs, the other. The former class will comprehend, *Stricture of the cardiac,—of the pyloric orifice; Tumors of the stomach of various kinds, particularly scirrhous; Ulceration, simple and carcinomatous.* The latter class, *Morbid conditions of the liver, particularly tumors;* and *enlargement and induration of the pancreas.*

Before we proceed farther, it may be well to collect them under one view, in order that they may serve as a standard of comparison whereby we may be enabled to discriminate them from the external signs of other abdominal maladies.

*The leading symptoms of central gastric stricture.* These, in the case which I have elsewhere recorded, are violent pain, soon (usually from twenty to thirty minutes) after taking food, signally aggravated by reclination on the *right* side, by ingestion of imperfectly masticated or fermentable aliment; by sudden atmospheric vicissitudes, by previous bodily fatigue, or depressing passions of the mind, generally succeeded by vomiting, and, when severe, seldom subsiding until the contents of the stomach were rejected; pain, seeming to begin in the very *centre* of the epigastric region; great emaciation; intervals, of longer or shorter duration, perfectly free from pain. No difficulty of deglutition; no induration or enlargement in the epigastrium; no hectic fever; no serous effusion, or other evident sign of hepatic lesion. In the case recorded by Blasius,* no morbid sign, except vomiting, is noted: in that from Morgagni,† the vomiting, which had existed twenty-four years, came on every day, *two hours* after dinner. The attendant pain completely subsided on rejection of the food, but was aggravated by efforts to retain it. The bowels were constipated. The gastric lesion was complicated with pericardiac adhesion, and cartilaginous induration of the aortic valves, denoted during life by an intermitting pulse. The contraction of the stomach does not appear to have been very strait. On the history delivered by Valsalva, I shall presently have occasion to comment.

We have now to concentrate, under distinct views, those external phænomena which commonly are observed in,

---

* Gerardus Blasius, *Observat. Med.* ix.

† Morgagni, *De Sedibus, &c.* Ep. xxx. art. 7.

and most strongly characterize, the two classes of abdominal lesions before indicated. In considering the different species, I shall observe the order in which they were mentioned, and illustrate those of more rare occurrence by examples borrowed from writers of undoubted authority.

### I. Lesions, which implicate the structure of the stomach itself.

1. *Stricture.*—A. *Stricture of the cardiac orifice. Leading symptoms. Difficulty or impracticability of deglutition,* according to the degree of stricture; vomiting *immediately after* ingestion of food; sense of pain and constriction in the epigastrium (commonly in the direction of the *cardiac orifice); often* hiccough. When the contraction is very great, the œsophagus dilates, and forms, with the contained aliment, a tumor between the layers of the posterior mediastinum, which compresses the lungs and heart. "I have opened the body of a man," says Portal,* "in whom the œsophagus was more capacious than the stomach. The latter was not larger than the stomach of an infant of two years. The cardiac orifice was so contracted, that it would scarcely admit a writing quill: its circumference was swollen, indurated, and unequally puckered. The parietes of the dilated portion of the œsophagus were greatly thickened." The same author adds, that, "independently of the causes which operate directly in contracting the cardiac orifice, there are some seated in the diaphragm: the opening left by that muscle for the passage of the œsophagus may be straitened by a tumor, as dissections have shewn." Cardiac stricture is of rare occurrence. We shall speak hereafter of cancer situated near this orifice.†—B. *Stricture of the pyloric orifice. Leading symptoms.* Vomiting, commonly in *one hour or longer,* after taking food; often pain referred to the region of the *pylorus.* Several cases of this kind may be seen in the works of Morgagni and Lieutaud. Dr. Baillie describes it as a rare malady.

2. *Tumors.*—Tumors, seated on the internal surface, differ considerably in size and character. Their *general symptoms* are pain and irritation of the stomach, more or less severe; vomiting; emaciation. But these symptoms

---

* Portal, *Cours d'Anatomie Medicale,* tome v. page 204.

† The rejection of the food, in stricture of the *cardiac orifice* of the stomach, is effected by a process very different from that of ordinary vomiting.

will vary according to the situation which the tumor occupies, its volume, and other circumstances. Seated near the cardia or pylorus, its symptoms will resemble those of cardiac or pyloric stricture. Exerting pressure on the biliary ducts or diaphragm, it may induce jaundice or hiccough; or, receiving an impulse from the action of the large vessels beneath, be mistaken for aortic or cœliac aneurism, as will be seen in a case which I shall shortly quote.* General dropsy is a common termination of it. But the diagnostic sign of gastric tumor, principally to be depended on, is *its detection by close examination of the epigastric region.* This is commonly practicable in its more advanced stages of developement. I know no means whereby the *scirrhous tumor* of the stomach may be distinguished from tumors of a different structure, except by the character of the attendant pain, and the constitutional and physiognomical peculiarities of the patient.

3. *Ulceration.*—A *Simple ulceration. Leading symptoms.* Vomiting of *purulent matter*, and discharge of it by stool; pain of the stomach, extending to the hypochondriac, umbilical, and renal regions; *hectic fever;* often violent cardialgia; sense of extreme heat and dryness in the throat; cough; jaundice. One of the chief diagnostic signs is, in this case, *vomiting of pus;* nor can too much attention be employed to ascertain correctly the purulent nature of the matter ejected; for, as I have before observed,† a morbid secretion, very strongly resembling pus, may sometimes be poured out by the vessels of the mucous membrane of a strictured stomach; and thus the observer, who contents himself with hasty or superficial investigation, will form an erroneous conclusion, as to the precise nature of the malady which he is called upon to treat.‡—B. *Car-*

* The following history, quoted from Lieutaud, presents a good example of gastric tumor: A melancholic complained perpetually of cardialgia, dysphagia, and watchfulness: on examining the epigastrium, a hard and circumscribed tumor was felt, two inches in breadth, and extending to the ensiform cartilage. Dyspnœa supervened: total inability to swallow; emaciation; prostration of strength; death.—*On dissection*, a scirrhous tumor, larger than a hen's egg, was found near (and closing) the cardiac orifice of the stomach; and another smaller in the inferior part of the organ, not far from the pylorus. *Hist. Anat. Med.* lib. i. obs. 91.—An interesting case and engraving of milt-like tumor of the stomach are given by Dr. Monro, *Morbid Anatomy of the Human Gullet, &c.* p. 164.

† See page 16 of this volume.

‡ Ulceration sometimes destroys all the coats of the stomach, and thus unnatural communications are formed between that organ and other cavities. Lieutaud found, on examining the body of a woman, long tormented

*cinomatous ulcer. Leading Symptoms.* Pain of the stomach, varying in different individuals, but commonly *violent and lancinating;* vomiting of a matter resembling *coffee-grounds, or soot mixed with a viscid fluid;* breath fœtid. The symptoms will be modified by the site and extent of the ulcerated surface, and other circumstances. If, however, there be much emaciation; if the cancerous tumor be situated near the pylorus or along the great arch of the stomach, and possess considerable volume, it may be felt, during life, on external examination.* The countenance of the patient generally becomes sallow, and assumes a striking peculiarity of expression as the disease advances. Chardel asserts, that cancerous affections of the stomach attack men much more frequently than women; and rarely occur till about the 40th year.† Having no case of my own to record in illustration of this dreadful malady, I shall extract, from a recently published number of a celebrated foreign Journal, a well-marked example of "ulcerated scirrhus of the cardia," and present it in an abridged form to my readers.

Monsieur R. aged 68, of a sanguineo-lymphatic temperament, strong constitution, and regular habits; subject to periodical catarrh, but otherwise healthy, was attacked, in September, 1807, with vomiting at the moment of swallowing food, whether solid or liquid. He was forced to pause suddenly; extreme sense of suffocation came on, with total extinction of voice. Rending pains behind the sternum, and intolerable faintness at heart, were also experienced. All these symptoms ceased on the ejection of glairy matter mingled with the little food which he had swallowed. He afterwards continued to eat with tolerable ease, and vomited again, once or more, without much difficulty. Exercise was productive of fatigue. For some time, the pulse continued calm; skin natural. The nights were passed comfortably; mouth clammy, but without ill taste; urine scanty, brick coloured, fœtid. Emaciation daily more visible; symptoms progressively aggravated;

---

with severe gastric pain and obstinate vomiting, the stomach adhering to the diaphragm, and an orifice of communication formed, by which the contents of the stomach had been effused into the thoracic cavity. The woman died of suffocation. *Hist. Anat. Med.* obs. 142.—And Portal, in a subject, with whose history he was unacquainted, observed an aperture, of ulcerative formation, between the stomach and cavity of the omentum. *Cours d'Anatomie Medicale,* tom. v. page 201.

* *Morbid Anatomy,* page 155.

† *Monographie des Dégenerations Skirrheuses de l'Estomac.*

deglutition completely impracticable; blood frequently thrown up in the violent efforts to vomit. Feet œdematous; urine more scanty, but inodorous; frequent discharge by the bowels of matter resembling that voided in *mœlena;* swelling of the hands; burning heat in the chest; constant agitation and efforts to vomit, the fluid ejected black or brownish, and mixed with shreds of membrane. Pulse variable; oppression; intolerable disgust. Tongue sometimes whitish, sometimes of a vermilion hue; frequent hiccough; profuse salivation. Latterly, liquids passed into the stomach with tolerable ease. Six days before death, perspiration took place on the head and breast, preceded by shivering. The œdema of the hands and feet subsided. Drowsiness; light delirium; anxiety. Cough and expectoration of a substance mixed with black filaments, and of a fluid resembling white of egg. On the 23d of December, the swelling of the arms returned; respiration became stertorous; the limbs cold. Death happened in the night.

*On Dissection,* the stomach was found smaller than common: it contained a puriform and blackish fluid. The mucous membrane of half the cardiac orifice was inflamed; the left lobe of the liver entirely gangrenous, and adherent to a carcinomatous ulcer, which was seated to the right of the cardia, and had utterly destroyed its parietes. The whole circumference of the ulcer was hard and scirrhous. The diameter of the cardiac orifice was not much diminished towards the œsophagus. The remainder of the stomach was sound. The spleen was shrunk, and the kidnies presented some traces of ulceration. The respiratory organs were free from disease.*

### II. Lesions which have their seat in the contiguous organs.

1. *The Liver.*—The affection of this organ, most likely to induce a train of phænomena resembling those of central gastric stricture, is *tumor or enlargement, of whatever kind, seated on its concave surface, and capable of exercising a mechanical influence upon the stomach.* Of this nature appears to have been the case recorded by Valsalva, and quoted by Morgagni, as an example of double stomach.† The *leading symptoms,* in this case, were pain and vomit-

* *Journal de Médecine, par Leroux.*

† *De Sedibus, &c.* Epist. xxxvi, art. 2.

ing after food; *induration and soreness of the right hypochondrium;* dyspnœa.

*On dissection,* the stomach was found greatly contracted at the centre by the pressure of the indurated left lobe of the liver; the right lobe greatly enlarged, and the peritonæum full of fluid. Now, it is highly important, that a consecutive or secondary contraction, of this nature, should be correctly distinguished from stricture induced by an original morbid alteration of the coats of the stomach; because the former might, under favourable circumstances, admit of palliation, or even cure, by mercury or other remedies calculated to reduce the bulk of the liver. *Tumor and induration, externally felt in the epigastric and right hypochondriac regions, with jaundice, dropsy, and other appearances denoting structural derangement of the liver,* would, I conceive, form here the diagnostic sign: for, until this organ is so much enlarged as to be felt externally, and to determine great constitutional derangement, its pressure upon the stomach can hardly be such, as to produce the peculiar phænomena of gastric stricture.

2. *The Pancreas.*—Vomiting, and pain in the epigastric region, are the *leading symptoms* of enlarged and indurated pancreas. Hence, this affection may readily be confounded with central gastric stricture; more particularly, as the pain and vomiting will commonly be aggravated *soon after ingestion* of food: for the pressure of the enlarged gland will more seriously incommode the stomach, in proportion as the latter is more distended. The case, to which these observations constitute a sequel, was frequently considered as one of morbid pancreas. The remote situation of the abdominal gland renders the path of diagnosis still more obscure: even when considerably increased in volume, it cannot be felt by external examination. Indeed, I know but of one circumstance, at all calculated to assist us in discriminating the gastric from the pancreatic lesion: it is remarked, in the narrative of the case to which I have just alluded, that the pain and vomiting were invariably aggravated by "*reclination on the right side.*" Now, if my explanation of the origin of this phænomenon be correct,* it must constitute one of the *essential* signs of central gastric stricture. But, from reasoning, I should not suppose that the same phænomenon is likely to occur in cases of pancreatic enlargement; and this inference is

* See Note, page 11 of this volume.

sanctioned, at least negatively, by the observations of all the pathological writers whom I have consulted on the subject.

Tumors growing from the inferior surface of the diaphragm, or formed by enlargement of the absorbent glands between the liver and stomach, might, if so situated as to compress the latter organ, produce effects very closely resembling those which result from central gastric stricture; but it is not my object to comprehend, in this sketch, *every* variety of abdominal lesion with which, from its attendant phænomena, this peculiar form of gastric contraction may be confounded. My observations must be necessarily restricted to those most strongly marked in character, and most frequent in occurrence.

*Recapitulation.*—It may be well, ere we conclude, to recapitulate the leading phænomena which serve to distinguish central stricture of the stomach from other lesions of the gastric and contiguous organs; and to collect, into one focus, all the feeble light which may be elicited from the preceding observations, on the diagnosis of abdominal maladies. In doing this, I shall adhere to the arrangement before instituted.

*Central stricture of the stomach* may then be distinguished

*From stricture of the cardiac orifice,* by the absence of *difficult deglutition;* by the rejection of the aliment *not immediately* following its ingestion; by the seat of the pain commonly referred to the centre of the epigastrium:

*From stricture of the pyloric orifice,* chiefly by the seat of the pain, which, in pyloric stricture, commonly affects the region of the pylorus; perhaps, also by the proportionately shorter interval which passes between the periods of ingestion and rejection of aliment: but this symptom is fallacious; for, in many cases of strictured pylorus, the food has not been retained by the stomach more than half an hour; while, in others, of central contraction, as in that which I have quoted from Morgagni, two hours have passed before the contents of the stomach were rejected by vomiting. *In general, however, vomiting will take place sooner after the reception of food, in central, than in pyloric stricture.* Correct discrimination of these two maladies must be exceedingly difficult:

*From tumors of the internal membrane,* by the absence of *swelling and induration in the epigastric region.* No gastric tumor would induce the peculiar symptoms of *central* stricture, unless it had acquired a volume, and occupied a site rendering it obvious to external examination:

*From simple ulceration,* by the absence of *pus* in the substances discharged per os et anum; by absence of *hectic fever:*

*From carcinomatous ulceration,* by the absence of *tumor and induration of the epigastrium;* of the *hectic fever;* of the *coffee-ground vomiting;* by the absence of those physiognomical and constitutional signs which denote the cancerous diathesis; and of the dreadful and peculiar pains, and rapidity of progress, which characterize the existence of cancer: The sex and age of the patient may also be admitted here as collateral evidence:

*From hepatic enlargement exercising pressure upon the stomach,* by the absence of *tumor and induration of the left lobe of the liver;* and of jaundice, dropsy, or other symptoms, indicating structural derangement of that organ:

And lastly, *From enlargement and induration of the pancreas,* by the *marked aggravation of the pain and vomiting experienced, in central stricture of the stomach, from reclination on the right side.* I know of no other sign capable of assisting in the discrimination of these two obscure and nearly allied forms of internal lesion.

Of emaciation, nothing needs to be said. It characterizes generally, more or less, the maladies which we have been considering, as well as all others of which vomiting constitutes an *essential* sign.

Having now, to the best of my ability, traced the parallel between central stricture of the stomach and other organic lesions, most closely resembling it in their external phænomena, I, perhaps, cannot more appropriately close this paper, than by the narrative of a case; which, while it demonstrates the occasional fallacy of the best established rules of diagnosis, and the imperfections of clinical observation unaided by anatomical research, may afford a salutary lesson to the inexperienced or self-confident practitioner, or to those who are in the habit of glancing at diseases with a hasty and superficial eye.

In May last, a man, aged 60, complained of pain in the left side of the abdomen, mid-way between the umbilicus and crest of the ilium. Otherwise he was healthy. Leeches relieved him for a time; but the pain soon returned in an aggravated form. Emaciation, weakness, distress of countenance, anorexia, constipation, succeeded. Nothing uncommon could be discovered in the painful part. At length, on more correct examination, a large tumor was discovered in the epigastric region, *with pulsations which seemed rather to distend than elevate it.* The case was now pronounced aneurism of the abdominal aorta.—Emaciation proceeded with rapid strides. The voice was lost. Great restlessness; the mouth clammy, without thirst. *No nausea or vomiting,* except that, some days before death, a quantity of fœtid, ill-tasted, blackish fluid was twice or thrice thrown up. *No fever.* The tumor, though more plainly felt, did not project externally: it caused a sense of constriction *rather than pain:* the pulsations became more perceptible. The stroke of the radial artery was feeble, *but slow and regular.* Life was terminated, by a hard struggle, on the 19th of August.

*Upon dissection,* the stomach was found adhering to the liver, pancreas, and parietes of the abdomen; and a *cancerous tumor* occupying its internal surface, from the duodenum to the insertion of the œsophagus. With the exception of a tumor situated near this, all the cancerous surface exhibited a pulpy, blackish, gangrene-like appearance; and the coats of the stomach were, at that part, an inch thick. On tearing asunder the adhesions which the stomach had formed to the liver and pancreas, two holes, of considerable size, were seen in the parietes of the former; but the continuity of surface had been preserved by the process of morbid adhesion; and thus effusion of the contents of the stomach into the abdominal cavity had been prevented. The surface of the pancreas was contaminated by the cancerous affection. The pyloric orifice, situated in the midst of the cancerous mass, was contracted by the thickening of the parietes of the stomach, and obstructed by numerous putrid fungi. The liver was voluminous, but otherwise healthy; the spleen small and readily lacerable. *The aorta, the cœliac trunk and its branches, were completely sound.**

Tamworth, March 8, 1816.

---

